# A Perspective on High-Intensity Interval Training for Performance and Health

**DOI:** 10.1007/s40279-023-01938-6

**Published:** 2023-10-07

**Authors:** Alexandra M. Coates, Michael J. Joyner, Jonathan P. Little, Andrew M. Jones, Martin J. Gibala

**Affiliations:** 1 Department of Kinesiology, McMaster University, 1280 Main St West, Hamilton, ON L8S 4K1 Canada; 2 Department of Anesthesiology and Perioperative Medicine, Mayo Clinic, Rochester, MN USA; 3 School of Health and Exercise Sciences, University of British Columbia, Kelowna, BC Canada; 4 Sport and Health Sciences, University of Exeter, Exeter, UK

## Abstract

Interval training is a simple concept that refers to repeated bouts of relatively hard work interspersed with recovery periods of easier work or rest. The method has been used by high-level athletes for over a century to improve performance in endurance-type sports and events such as middle- and long-distance running. The concept of interval training to improve health, including in a rehabilitative context or when practiced by individuals who are relatively inactive or deconditioned, has also been advanced for decades. An important issue that affects the interpretation and application of interval training is the lack of standardized terminology. This particularly relates to the classification of intensity. There is no common definition of the term “high-intensity interval training” (HIIT) despite its widespread use. We contend that in a performance context, HIIT can be characterized as intermittent exercise bouts performed above the heavy-intensity domain. This categorization of HIIT is primarily encompassed by the severe-intensity domain. It is demarcated by indicators that principally include the critical power or critical speed, or other indices, including the second lactate threshold, maximal lactate steady state, or lactate turnpoint. In a health context, we contend that HIIT can be characterized as intermittent exercise bouts performed above moderate intensity. This categorization of HIIT is primarily encompassed by the classification of vigorous intensity. It is demarcated by various indicators related to perceived exertion, oxygen uptake, or heart rate as defined in authoritative public health and exercise prescription guidelines. A particularly intense variant of HIIT commonly termed “sprint interval training” can be distinguished as repeated bouts performed with near-maximal to “all out” effort. This characterization coincides with the highest intensity classification identified in training zone models or exercise prescription guidelines, including the extreme-intensity domain, anaerobic speed reserve, or near-maximal to maximal intensity classification. HIIT is considered an essential training component for the enhancement of athletic performance, but the optimal intensity distribution and specific HIIT prescription for endurance athletes is unclear. HIIT is also a viable method to improve cardiorespiratory fitness and other health-related indices in people who are insufficiently active, including those with cardiometabolic diseases. Research is needed to clarify responses to different HIIT strategies using robust study designs that employ best practices. We offer a perspective on the topic of HIIT for performance and health, including a conceptual framework that builds on the work of others and outlines how the method can be defined and operationalized within each context.

## Key Points

**Table:** 

Interval training is a simple concept that refers to repeated bouts of relatively hard work interspersed with recovery periods of easier work or rest.
There is no common definition of “high-intensity interval training” (HIIT) despite its widespread use. In a performance context, HIIT can be characterized as intermittent bouts performed above the heavy-intensity domain. This characterization of HIIT is primarily encompassed by the severe-intensity domain. In a health context, HIIT can be characterized as intermittent bouts performed above moderate intensity. This characterization of HIIT is primarily encompassed by the classification of vigorous intensity.
Sprint interval training (SIT) constitutes a particularly intense variant of HIIT that can be distinguished as repeated bouts performed with near-maximal to “all out” effort. This characterization coincides with the highest intensity classification identified in training zone models or exercise prescription guidelines, including the extreme-intensity domain, anaerobic speed reserve, or near-maximal to maximal intensity classification.
In an endurance-sport context, there is little question that HIIT is an essential component of a comprehensive training program, but the specific training intensity distribution and optimal types of interval training sessions to enhance performance are still unclear.
From a health perspective and given the strong inverse relationship between cardiorespiratory fitness and morbidity and mortality, research is warranted to identify optimal HIIT strategies in different populations using robust study designs.

## Introduction

Interval training is a simple concept that can be defined as repeated bouts of relatively hard work interspersed with recovery periods of easier work or rest [1]. The method is commonly viewed in the context of athletic performance and has been a staple of training programs for high-level endurance athletes for over a century [2, 3]. It is deemed critical for success in sports and events such as middle- and long-distance running [2, 4], cycling [5], swimming [6], rowing [7], and cross-country skiing [8]. A central tenet of interval training in an athletic context is to accumulate a greater volume of work at a higher intensity than could be achieved through continuous work at a fixed intensity [9]. This in turn is believed to potentiate physiological responses and facilitate the capacity to maintain a higher work rate and enhance fatigue resistance during competition [3, 10]. While interval training is widely regarded as an essential component to optimize performance, the high overall volume of training that is typically practiced by endurance athletes requires that the total time spent at a high intensity be managed to reduce the risk of overreaching, injury, and illness [11, 12].

The concept of interval training to improve health has also been advanced for decades. This includes application of the method in relatively inactive individuals, older adults, or a rehabilitative context in patients with specific conditions [13–15]. Early proponents saw value in the approach as compared to traditional continuous training. It was noted that intervals allowed “the beginner (to) complete more work with less fatigue” [15], and even in very deconditioned patients, the “appropriate choice of exercise and recovery intervals [means] substantial cardiac training can be achieved” [14]. Research on the physiological basis of interval training to improve health, including so-called “low-volume” approaches that do not require substantial time commitment [16, 17], has increased significantly over the last two decades. This has coincided with considerable interest in the topic from an applied perspective, as evidenced by annual worldwide surveys of fitness trends [18, 19].

This article offers a brief a perspective on the topic of interval training for performance and health. The term “performance” is used primarily in the context of exercise training for athletic competition. The term “health” is used in the context of habitual exercise intended to maintain or enhance physical fitness and reduce disease risk. Our focus is on endurance-type sports and events, as commonly considered in training models for endurance athletes [20–23], and aerobic/cardiorespiratory physical activity as defined in authoritative public health and exercise prescription guidelines [24–26]. While the intermittent work bouts characteristic of interval training are relatively short and discontinuous, with each typically lasting from a few seconds to several minutes, the method is often performed with the goal of enhancing endurance-type performance or aerobic/cardiorespiratory capacity. Most of the energy during intermittent exercise is derived from aerobic metabolism, including during repeated “all out” sprints [27], and even brief, intense interval training is associated with an enhanced capacity for aerobic energy provision [28, 29]. Building on the work of others [20–26], we present a conceptual framework that outlines how “high-intensity interval training” (HIIT), and a particularly intense variant called “sprint interval training” (SIT), can be defined and operationalized within each context. Specific issues that are considered include the optimal intensity distribution and type of interval training in highly trained athletes [30] and interval training strategies to increase cardiorespiratory fitness with a focus on individuals who are apparently healthy.

## Framing the Issue: What is “High-Intensity” Interval Training?

An important issue that impacts the discussion of interval training in both a performance and health context is the lack of standardized terminology. This particularly relates to the classification of intensity. Various stakeholders do not “speak the same language,” and definitions of absolute and relative intensity vary across public health agencies, exercise scientists, clinicians, practitioners, coaches, and athletes [20, 21, 23, 25, 26].

**Fig. 1:**
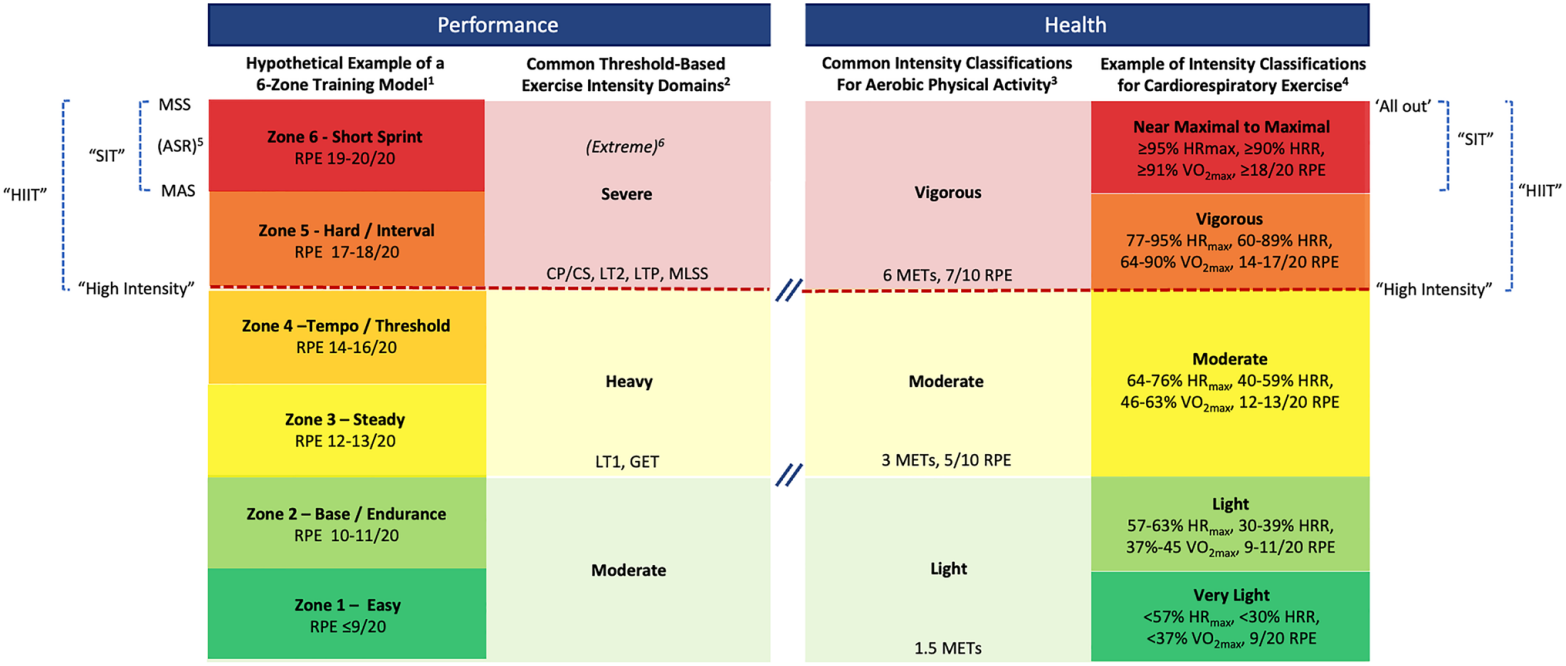
A conceptual framework for application of interval training in performance and health contexts. ^1^Authors’ example modeled after common frameworks including elements from Seiler [20]; Casado et al. [21]; and Jamnick et al. [23]. ^2^Common three-domain classification based on work rate or physiological indicators [22, 31, 32]. ^3^World Health Organization 2020 guidelines on physical activity [25]. ^4^American College of Sports Medicine’s guidelines for exercise testing and prescription, 11th ed. [26]. ^5^Anaerobic speed reserve [39]. ^6^Extreme-intensity domain [22, 35]. *ASR* anaerobic speed reserve, *CP/CS* critical power/critical speed, *GET* gas exchange threshold, *HIIT* high-intensity interval training, *HR*_*max*_ maximal heart rate, *HRR* heart rate reserve, *LT1* first lactate threshold, *LT2* second lactate threshold, *MAS* maximal aerobic speed, *MET* metabolic equivalent of task, *MLSS* maximal lactate steady state, *MSS* maximal sprint speed, *RPE* rating of perceived exertion (out of either 10 or 20 depending on the scale), *SIT* sprint interval training, $\dot{V}{\text{O}}_{2\max }$ maximal oxygen uptake

A fundamental three-domain classification scheme is common in a performance context (Fig. 1). This characterization is based on indicators that mark the transitions between moderate, heavy, and severe exercise intensity [22, 31, 32]. The first lactate threshold or gas exchange threshold (GET) commonly denotes the boundary between moderate and heavy domains. This marks the point where blood lactate begins to accumulate above baseline values and the oxygen uptake ($\dot{V}{\text{O}}_{2}$) slow component is elicited [33]. The determination of critical power or critical speed (sometimes called the critical $\dot{V}{\text{O}}_{2}$) marks the boundary between the heavy and severe domains [32, 33]. This reflects the highest “sustainable” intensity, above which there is a marked increase in non-oxidative metabolism associated with rapid exercise intolerance [32]. Other indicators that denote the boundary between the heavy and severe domains include the second lactate threshold, maximal lactate steady state (MLSS) or the lactate turnpoint [33, 34]. In some models, there is a fourth extreme*-*intensity domain that involves an effort level of such intensity that maximal oxygen uptake ($\dot{V}{\text{O}}_{2\max }$) cannot be achieved if $\dot{V}{\text{O}}_{2}$ is measured during exercise, and the bouts are necessarily of short duration, typically less than 2 min [22, 35].

Beyond the well-accepted three-domain framework, greater nuance is often sought for exercise prescription in high-level or elite sport. Many such models have been proposed for endurance training, including those comprised of five [23, 36], six [21], or seven [37] distinct zones. These zones are typically distinguished by various metrics, including those related to rating of perceived exertion (RPE), percentage of maximal heart rate (HR_max_) or $\dot{V}{\text{O}}_{2\max }$, or blood lactate levels [21, 36, 37]. We have included six zones by way of example in Fig. 1, such that each of the three main domains are broken into two zones that are demarcated by RPE. Additional descriptors related to HR_max_, $\dot{V}{\text{O}}_{2\max }$, or blood lactate could be applied on an individual basis derived from sport-specific testing [20].

In a health context, the basic intensity classifications of aerobic physical activity by authoritative agencies, including the World Health Organization (WHO), are light, moderate, and vigorous [25]. These three categories are typically distinguished by indicators based on metabolic equivalents (METs) or RPE on a 10-point scale. Exercise testing and prescription guidelines from other authoritative agencies typically incorporate additional categories or levels, with boundaries anchored to percentages of HR_max_, heart rate reserve, or $\dot{V}{\text{O}}_{2\max }$, in addition to RPE and METs. For example, the American College of Sports Medicine (ACSM) defines light, moderate, and vigorous intensity based on percentages of heart rate reserve, $\dot{V}{\text{O}}_{2\max }$, METs, or RPE on a 20-point scale [26]. The ACSM includes the additional categories of very light and near-maximal to maximal, with corresponding relative and absolute thresholds that fall below and above, respectively, the other three categories (Fig. 1).

There is no common definition of the term HIIT despite its widespread use. As recently highlighted and discussed by others [38], this creates confusion and interpretational challenges. We contend that in a performance context, HIIT can be characterized as intermittent bouts performed above the heavy-intensity domain. As noted, this is demarcated by indicators that primarily include the critical power or critical speed, or other indices, including the second lactate threshold, MLSS, or lactate turnpoint. This characterization of HIIT is primarily encompassed by the severe-intensity domain. The relatively high work rate required precludes sustained efforts and thus an intermittent approach permits greater time to be accumulated at the desired work rate [35]. This is conceptually consistent with how previous researchers have generally defined “high-intensity” training in a performance context [10, 20, 21]. A particularly intense variant of HIIT, SIT, can be distinguished as repeated bouts performed with near-maximal to “all out” effort. This characterization coincides with the highest intensity classification included in some training zone models, including the extreme-intensity domain [22, 35] or anaerobic speed reserve, which constitutes work rates between maximal aerobic speed or power and maximal sprint speed/power [39].

**Fig. 2:**
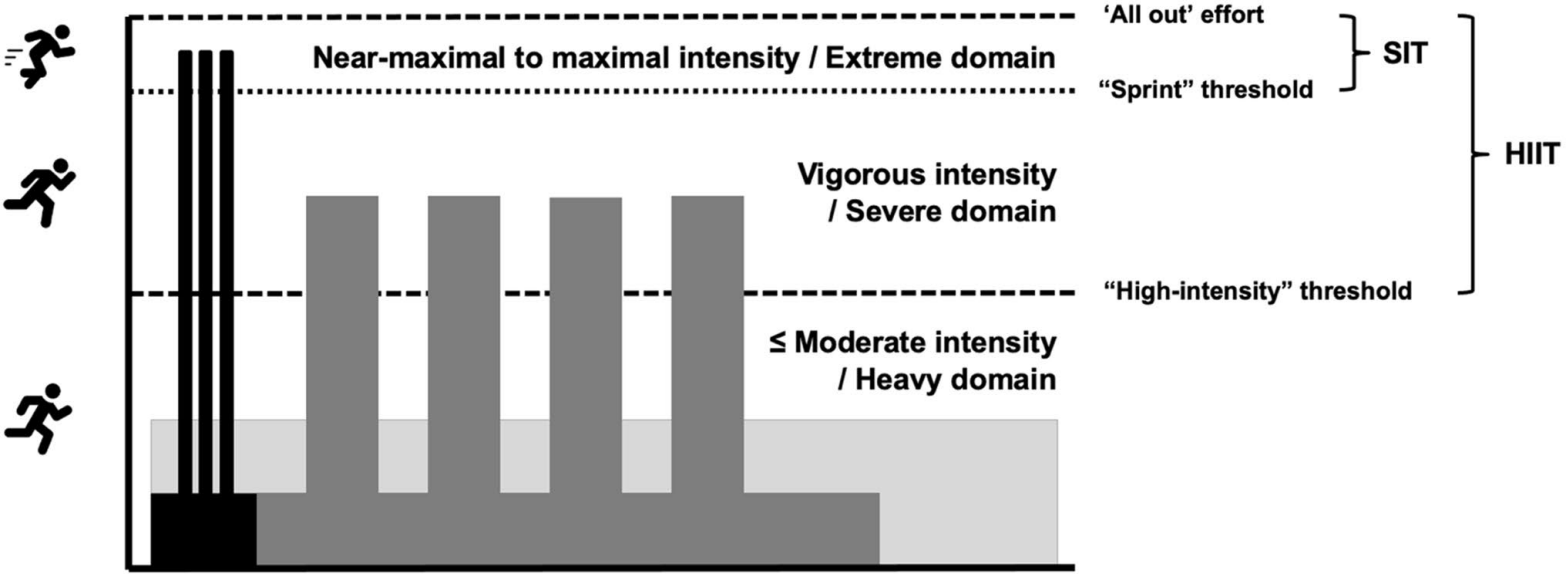
Simplified depiction of sample high-intensity interval training (HIIT) and sprint interval training (SIT) protocols with reference to thresholds demarcated in common domain-based training models and physical activity and exercise intensity classifications [22, 24–26, 31, 32]. Icons made by Prosymbols Premium (top left) and Freepik (middle and bottom left) from Flatiron (www.flaticon.com/free-icons/trail-running; www.flaticon.com/free-icons/run; www.flaticon.com/free-icons/chase)

HIIT is even less well defined in a health context. Our own work has regrettably contributed to the nebulous depiction; for example, an early review of the physiological responses to “HIIT” [40] was based mainly on studies that used “SIT” interventions. This contributed in part to fostering the erroneous notion that HIIT is mainly characterized by activities that involve very high intensity, near-maximal, or “all out” efforts. As the field has evolved, so too has the terminology, and efforts have been made to distinguish responses to different types of interval training. Weston and colleagues [41] defined HIIT as a “target intensity between 80% [and] 100% peak heart rate” and differentiated SIT as a “target intensity ≥ 100% $\dot{V}{\text{O}}_{2\max }$.” We and others have employed similar terminology to characterize responses to these two broad types of interval training, as distinguished from traditional moderate-intensity continuous training (MICT) [42]. Building on this framework and broadening it to include multiple indicators as opposed to a single metric, we contend that HIIT can be characterized as intermittent bouts performed above moderate intensity. This characterization primarily encompasses the classification of vigorous intensity demarcated by indicators related to perceived exertion, $\dot{V}{\text{O}}_{2}$, or heart rate as defined in authoritative public health and exercise prescription guidelines [24–26]. Analogous to a performance context, SIT can be considered an intense variant of HIIT and distinguished as repeated bouts performed with near-maximal to “all out” effort that fall within the highest intensity classification included in some guidelines [24–26] (Fig. 2).

In recommending an operational framework for HIIT in a health context that is based on “traditional” indicators, it is recognized that expressing intensity in this manner (and particularly related to percentages of HR_max_ or $\dot{V}{\text{O}}_{2\max }$) may not be optimal. This point has been made by others [43], and recent studies have shown that variability in exercise tolerance may be reduced, and exercise training responses be more homogenous or strongly associated, when intensity is expressed relative to “physiological” thresholds such as critical power or speed rather than anchors such as $\dot{V}{\text{O}}_{2\max }$ [44, 45]. RPE is a particularly useful indicator of HIIT intensity, including for its convenience and wide applicability, although this marker also has limitations, especially when interval work bouts are quite brief and performed at very high intensities. The conceptual framework proposed here also does not preclude the application of physiological thresholds or performance-based indicators of HIIT in other contexts such as training studies that are focused on health-related responses.

## High-Intensity Interval Training for Performance

Current-day, high-level endurance athletes typically employ a training intensity distribution that involves ~ 70–90% of training volume in the moderate-intensity domain and ~ 10–30% of the training volume at higher intensities in the heavy- or severe-intensity domains [4, 46, 47]. It is contested as to whether the ~ 20% of higher intensity training should be distributed in a pyramidal fashion where there is decreasing training volume accrued from the heavy to severe domains, or in a polarized fashion where the remaining ~ 20% is performed primarily in the severe domain [4, 11, 48]. So-called “threshold training” is a third potential approach in which > 35% of the training volume falls in the heavy-intensity domain [49]; however, this distribution has occasionally been shown to be inferior to polarized or pyramidal intensity distributions for improving endurance performance [47, 50, 51]. Despite this, the best Kenyan marathon runners in the world are reported to follow a threshold training distribution during the specific preparatory phase leading into marathon competition [51, 52], which may be particular to the physiological demands of marathon racing [37, 51]. Elite swimmers may also follow threshold training distributions as interval training makes up most daily sessions. A greater amount of interval training is likely used in swimming because most training sessions are supervised, it allows more athletes to be accommodated in a limited pool space, and the orthopedic stress associated with swimming is less than with running [6, 53]. There is likely no single optimal training intensity distribution for all endurance sports and events, but rather determination of the optimal individualized and sport-specific periodization of intensity distribution may be the next step in this field. A systematic review of highly trained/elite distance runners found most training phases involved a pyramidal training intensity distribution; however, a shift towards a polarized approach was often employed during the competition phase [4]. Future work should examine this periodization of training intensity distribution as it relates to highly trained or elite endurance performance across different events.

All training intensities, ranging from prolonged, continuous sessions in the moderate domain to repeated “all out” sprint interval sessions, can improve endurance performance, provided the training is balanced within a wider program specific to the demands of the sport [2, 21]. The optimal type of HIIT to enhance endurance performance in highly trained athletes who have little room for additional physiological improvement is still unclear. It has been suggested that a greater accumulated training time close to $\dot{V}{\text{O}}_{2\max }$ (typically ≥ 90% of $\dot{V}{\text{O}}_{2\max }$) is ideal for maximizing aerobic adaptations, particularly in highly trained individuals [2, 54–57]. A seminal study by Billat et al. demonstrated that 30-s intervals performed until exhaustion at the minimal velocity to elicit $\dot{V}{\text{O}}_{2\max }$ ($v\dot{V}{\text{O}}_{2\max }$), with 30 s of active recovery at 50% of $v\dot{V}{\text{O}}_{2\max }$, accumulated more than double the time spent at $\dot{V}{\text{O}}_{2\max }$ when compared to continuous running to exhaustion above critical speed in trained runners [9]. Other studies have since examined variations of interval length [58–61], intensities [62], work-to-rest ratios [61, 63], and pacing strategies [64–68] to optimize the accumulated time ≥ 90% of $\dot{V}{\text{O}}_{2\max }$. However, these investigations were rarely performed in highly trained or elite athletes, who have faster $\dot{V}{\text{O}}_{2}$ kinetics [69], a reduced $\dot{V}{\text{O}}_{2}$ slow component in the severe-intensity exercise domain [70], and faster recovery rates between intervals [71, 72] compared to lesser trained individuals. Further, many of these studies examined the time accumulated ≥ 90% of $\dot{V}{\text{O}}_{2\max }$ in workouts performed to exhaustion [9, 62, 64, 65, 67, 68], which is not practical for high-performance athletes who rarely train to failure. Nevertheless, Rønnestad et al. demonstrated that when training programs were matched for total volume and intensity, 3 weeks of repeated sprint interval sessions [3 sets (13 × 30-s intervals at maximal sustainable intensity, with 15 s recovery) with 3 min between sets] improved $\dot{V}{\text{O}}_{2\max }$, maximal aerobic power, and 20-min cycling power in elite male cyclists (mean $\dot{V}{\text{O}}_{2\max }$ of 73 ± 4 mL kg^−1^ min^−1^), and this did not occur with RPE-matched longer interval sessions (4 × 5-min intervals at maximal sustainable intensity with 2.5 min recovery) [73]. While this type of high-volume repeated, short-interval training has been used for decades [2], it may not be commonly employed in endurance training programs [4, 7, 37, 74], and thus might represent a stimulus for further performance enhancement in this population.

When prescribing longer (~ 5-min) intervals in highly trained athletes, “fast-start” and/or variable-speed intervals may allow for greater accumulated time ≥ 90% of $\dot{V}{\text{O}}_{2\max }$ compared to constant-speed intervals [75, 76]. One investigation in cross-country skiers demonstrated that 5 × 5-min intervals above the second lactate threshold with 3 min of recovery performed with either a fast start (2 min at maximal aerobic speed) or variable speed (3 × 40-s surges at maximal aerobic speed) elicited greater time ≥ 90% of $\dot{V}{\text{O}}_{2\max }$ compared to constant-speed intervals of similar mean interval speeds [76]. Of interest, the sessional RPE was similar across conditions, demonstrating the feasibility of this approach for elevating the metabolic stimulus of these longer-interval sessions [76].

Finally, SIT is a relatively understudied technique that may improve endurance performance in athletes with little room for further adaptation [77–79]. Runners often use short sprints or strides following warm-up or at the end of workouts with the intention of training high-velocity movement patterns but not accumulating fatigue [37]. The addition of short 30-s sprint intervals to long rides has been demonstrated to be well-tolerated by elite cyclists and may enhance fatigue-resistance/durability [79]. Importantly, durability, or the time of onset and magnitude of deterioration in physiological performance characteristics during prolonged exercise, may be a critical predictor of performance in endurance sport [80–82]. Almquist et al. [79] demonstrated that the addition of maximal sprint intervals during a 2-week high-volume cycling training camp allowed for the maintenance of gross economy in a semi-fatigued state compared to reductions in gross economy in the non-sprint group, suggesting durability was improved with the sprint training [79]. However, as the addition of maximal-intensity intervals with the maintenance of typical training load is a technique used to drive underperformance (overreaching) [83], this type of training should be considered in the context of the desired training-intensity distribution and overall training load, and not simply prescribed in addition to regular training.

Another method of SIT prescription uses a percentage of the anaerobic speed reserve to dictate intensity, rather than a percentage of maximal aerobic speed or maximal aerobic power, $\dot{V}{\text{O}}_{2\max }$, or v $\dot{V}{\text{O}}_{2\max }$. In this model, the anaerobic speed reserve represents the difference between maximal sprint speed/power and maximal aerobic speed/power [39], and can differ greatly between athletes of similar caliber within the same event [84]. While the use of a percentage of anaerobic speed reserve to prescribe SIT intensities has not been investigated in highly trained endurance athletes, there is evidence that this method reduces interindividual variability in the physiological response to the workout and may increase time spent ≥ 90% of $\dot{V}{\text{O}}_{2\max }$ [85–87]. As such, with appropriate programming, all-out sprint intervals may represent an effective method for further performance enhancement in highly trained and elite athletes [88].

Interventional training studies involving highly trained athletes are limited, and as such, our understanding of optimal HIIT (or SIT) prescription for endurance performance is based largely on what is commonly practiced. Recent studies by Rønnestad et al. are a reminder of the feasibility and utility of performing randomized controlled training studies in elite participants [73, 76]. Further experimental investigations are required to determine ideal interval types for performance enhancement in this population. There are very few studies on highly trained or elite female athletes, and given that sex differences could affect the response to interval training [89], it is also imperative that more female athletes be included in future research of this kind.

## High-Intensity Interval Training for Health

A major emphasis of research on interval training for health, which will be the primary focus of this section, is cardiorespiratory fitness as best objectively determined by a $\dot{V}{\text{O}}_{2\max }$ test. This is owing to both the routine assessment of $\dot{V}{\text{O}}_{2\max }$, making it arguably the most measured variable in HIIT studies, and the importance of cardiorespiratory fitness in terms of mortality and morbidity risk [90, 91]. A recent meta-analysis, based on 37 studies with objective measures of cardiorespiratory fitness in over 2 million adults, found the relative risk for all-cause mortality was reduced by 11% for every 1 MET increase in cardiorespiratory fitness independent of age, biological sex, and duration of follow-up [92]. As recently reviewed by Ross and Myers [91], heritability may account for up to ~ 50% of the individual variation in the response of cardiorespiratory fitness to exercise training, but it is firmly established that cardiorespiratory fitness increases in response to regular physical activity in most adults. Randomized controlled trials considering the interaction between exercise intensity and exercise amount (typically determined by estimated energy expended) have suggested that intensity is the strongest driver of the increase in cardiorespiratory fitness [93–95], but such trials have involved continuous exercise interventions.

Many studies have compared the cardiorespiratory fitness response between MICT and HIIT of various types, employing both “matched” and “non-matched” approaches. Such comparisons are usually based on some measure or estimate of total energy expenditure (e.g., based on $\dot{V}{\text{O}}_{2}$), or less commonly on a measure of total work (e.g., mean power output). The most comprehensive study to date of the cardiorespiratory response to HIIT and MICT matched for estimated energy expenditure is Generation 100 [96]. This trial randomized over 1500 older participants (mean age ~ 73 years) to perform two sessions weekly of HIIT (~ 90% of peak heart rate), MICT (~ 70% of peak heart rate), or to follow national guidelines for physical activity (effectively combined HIIT and MICT) for 5 years. The increase in peak heart rate after 1, 3, and 5 years of the interventions was higher in HIIT compared to MICT and the combined group. Unlike Generation 100, a general limitation of many comparative studies is that they are relatively small and short term, with interventions often lasting ≤ 6–12 weeks [91]. Systematic reviews and meta-analyses based on these smaller, shorter studies have concluded that HIIT can elicit increases in cardiorespiratory fitness comparable to MICT despite a lower total exercise volume [97, 98], and the increase in cardiorespiratory fitness is greater after HIIT compared to MICT when exercise volume is matched [98, 99]. Our focus here is on people who are apparently healthy, but systematic reviews and meta-analyses including individuals with cardiovascular disease [41, 100], hypertension [101], and type 2 diabetes [102] have also concluded that the increase in cardiorespiratory fitness after HIIT is superior to MICT when total work is matched. Such findings are not universal [103, 104], and a recent review [105] highlighted methodological concerns with many comparative studies in this field. The main concerns are related to research design limitations and an unclear risk of bias owing to poor reporting quality in studies comparing interval and continuous training. These authors also noted that such shortcomings are not unique to the field of interval training, and they emphasized that the best practices outlined in their review [105] are applicable to all disciplines within exercise and sports medicine research.

Irrespective of how HIIT compares to MICT, and specific details on the most appropriate way to make such comparisons, an important question that warrants further investigation is whether simple, practical applications of interval training constitute a sufficient stimulus to increase cardiorespiratory fitness and in turn reduce the risk for morbidity and mortality. With respect to the physiological basis of responsiveness, there is good evidence that interventions requiring a total time commitment of ≤ 15 min, including warm-up and cooldown and performed at least thrice weekly for 6 weeks, increase $\dot{V}{\text{O}}_{2\max }$ by ~ 1 MET [29]. Many of these studies have employed SIT as commonly understood and defined here, but there are examples of less intense HIIT protocols that elicit similar responses over the short term [106, 107]. The precise mechanisms remain to be elucidated but seemingly include an enhanced capacity for skeletal muscle oxygen diffusing capacity and oxygen utilization, as well as potentially augmented central delivery of oxygen [29]. Other practical and relatively time-efficient applications of the method that have been shown to increase $\dot{V}{\text{O}}_{2\max }$ include activities such as brief vigorous stair climbing [108], bodyweight style exercise that incorporates aerobic and resistance exercise (sometimes called “high-intensity functional training”) [109, 110], and “exercise snacks” in which very short (≤ 1-min) bouts of vigorous-intensity activity are performed periodically throughout the day [111]. The methodological, risk of bias, and reporting quality concerns noted above apply similarly to this research, and studies to date involve a relatively small number of participants and may be underpowered to assess meaningful differences in specific outcomes. Additional work is warranted to advance this area.

Another emerging area of interest is the potential to employ preoperative HIIT as a strategy to improve cardiorespiratory fitness and improve surgical outcomes. A recent systematic review and meta-analysis [112] considered randomized clinical trials and prospective cohort studies with HIIT protocols in adult patients undergoing major surgery. Based on 12 included studies and a total of 832 patients, the analysis found several positive associations for HIIT when compared with standard care on cardiorespiratory fitness (measured directly from a $\dot{V}{\text{O}}_{2\max }$ test or estimated from surrogate measures such as a 6-min walk test or peak power output) and postoperative outcomes including complications, length of stay in hospital, and quality of life. The analysis showed a high degree of heterogeneity in study outcomes and an overall low risk of bias. These findings suggest that preoperative HIIT may improve cardiorespiratory fitness and reduce postoperative complications. Another recent systematic review and meta-analysis provided further support for HIIT in the clinical management of important cardiometabolic health risk factors in addition to cardiorespiratory fitness (e.g., systolic and diastolic blood pressure, resting heart rate, stroke volume, and left ventricular ejection fraction) [113]. Improvements were also observed in parameters of body composition, lipids, fasting insulin, and anti-inflammatory changes via reductions in high-sensitivity C-reactive protein.

Some have questioned whether HIIT is a feasible option to improve health [114]. There is a tendency in such critiques to position HIIT as requiring a level of effort that is unpalatable or potentially unsafe for most people [114]. Such criticisms are seemingly not an indictment of HIIT per se (or at least only of HIIT) but rather a general dismissal of the potential adoption of the upper intensity range of physical activity recommendations and exercise prescription guidelines from authoritative agencies, including the WHO and ACSM, that broadly advocate moderate and/or vigorous intensity for most adults [24–26].

The potential benefit of even small amounts of vigorous physical activity on health was recently shown by Stamatakis et al. [115]. These authors examined the association between “vigorous intermittent lifestyle physical activity” (VILPA) and all-cause cardiovascular disease and cancer mortality over an average follow-up of almost 7 years in over 25,000 non-exercisers with a mean age of 62 years in the UK Biobank. VILPA refers to brief intermittent bursts of vigorous-intensity physical activity embedded into everyday life rather than performed as structured leisure time exercise, such as stair climbing or carrying children or groceries for short distances [116]. The median duration of 4.4 VILPA min/day was associated with a 26–30% reduction in all-cause and cancer mortality and 32–34% reduction in cardiovascular disease mortality risk. These findings highlight the potential value of brief and sporadic bouts of physical activity of higher intensity, performed outside the structured exercise domain, for promoting health. The authors concluded that future trials and device-based cohort studies should investigate the potential of VILPA as a time-efficient and potentially effective intervention for physically inactive and unfit adults.

## Conclusions

HIIT is a common component in the training prescription for high-level athletes. The potential application of HIIT for health in less trained individuals is also not novel but is becoming increasingly recognized. In an endurance sport context, there is little question that HIIT is an essential component of a comprehensive training program, but the specific training intensity distribution and optimal types of interval training sessions to enhance performance are still unclear. It is likely that the optimal training intensity distribution will vary by sport and by the individual athlete, and this also needs to be periodized across a macrocycle. HIIT optimization is an area of research that may enhance current endurance sport performance, with several emerging techniques showing promise for improving performance in highly trained and elite athletes. HIIT optimization is also required in a health context. Given the strong, inverse relationship between cardiorespiratory fitness and morbidity and mortality, research is warranted to identify the most effective HIIT strategies in various populations using robust study designs. The mechanistic basis of HIIT responses, and why the method may facilitate greater improvements specific to performance and health markers, is beyond the scope of this review but also warrants further investigation. This includes the fundamental question of whether differential responses between HIIT and MICT are related to the intrinsic alternating pattern of higher- and lower-intensity efforts or mainly the higher-intensity work per se. Some research shows that HIIT can elicit larger improvements in selected physiological markers related to oxygen delivery and utilization as compared to a matched volume of MICT [117–119]. Most of this work has employed active but not well-trained individuals, and the physiological basis of responsiveness in highly trained individuals who already have a well-developed capacity for aerobic energy metabolism is likely different [120, 121].
